# A Prognostic Analysis of Male Breast Cancer (MBC) Compared with Post-Menopausal Female Breast Cancer (FBC)

**DOI:** 10.1371/journal.pone.0136670

**Published:** 2015-08-27

**Authors:** Xing-Fei Yu, Hong-Jian Yang, Yang Yu, De-Hong Zou, Lu-Lu Miao

**Affiliations:** 1 Department of Breast Tumor Surgery, Zhejiang Provincial Cancer Hospital, Hangzhou, Zhejiang Province, P.R. China; 2 Department of Medical Oncology, Zhejiang Provincial Cancer Hospital, Hangzhou, Zhejiang Province, P.R. China; University of North Carolina School of Medicine, UNITED STATES

## Abstract

**Background:**

Male breast cancer (MBC) is known to be rare compared with female breast cancer (FBC) and to account for only 1% of all breast cancers. To date, male patients diagnosed with breast cancer are normally treated based on the guidelines for FBC. Specifically, studies have found that diagnosing and treating MBC patients under the guidelines for the treatment of post-menopausal FBC are more favorable than are those of pre/peri-menopausal FBC from a physiological perspective because MBC and post-menopausal FBC patients show high estrogen receptor (ER) expression in the tumor and low estrogen expression in the body. In this medical study, we aimed to examine whether MBC actually has the same prognosis as post-menopausal FBC.

**Method:**

We identified MBC patients who were diagnosed as operable and who completed clinical treatment and we used follow-up data that were collected from January 2001 to January 2011. Each MBC patient was paired with four FBC patients who were diagnosed within the same period (two were pre/peri-menopausal, and two were post-menopausal). We compared disease-free survival (DFS) and overall survival (OS) among three groups, i.e., pre/peri-menopausal FBC (group A), post-menopausal FBC (group B) and MBC (group M), using the Kaplan-Meier method and a Cox proportional hazards regression model. We also evaluated the clinical characteristics of breast cancer patients using t-tests and chi-square tests. We used ten consecutive years of data that were collected at Zhejiang Provincial Cancer Hospital.

**Results:**

We identified 91 MBC cases for group M, 182 FBC cases for group A and 182 FBC cases for group B. The median follow-up period was 112 months. MBC cases were much more frequently ER positive than those of group A and group B (p<0.01); a similar trend was also found for progesterone (PR)-positive cases (p<0.01). The MBC group showed much lower human epidermal growth factor receptor-2 (HER2) expression than did the other groups (p<0.01). The 10-year OS rates were 79.1% for group M (72/91), 79.1% (144/182) for group A, and 87.9% (160/182) for group B, log-rank test indicated that group M had similar mean OS time as group A and group B (GourpM vs group A: p = 0.709; group M vs group B: p = 0.042). The Cox proportional hazards regression model indicated that pre/peri-menopausal FBC had similar DFS (hazard ratio (HR) = 0.706, p = 0.262) and OS (HR = 1.029, p = 0.941) values compared with MBC, whereas post-menopausal FBC had higher DFS (HR = 0.454, p = 0.004) and OS (HR = 0.353, p = 0.003) values than did MBC.

**Conclusion:**

Based on this study, we can conclude that MBC displayed higher ER- and PR-positive expression and lower HER2-positive expression than both post-menopausal and pre/peri-menopausal FBC. However, the DFS and OS values of MBC were similar to those of pre/peri-menopausal FBC and were worse than were those of post-menopausal FBC.

## Introduction

Few studies over the years have shown evidence of increasing numbers of male breast cancer (MBC) patients in western and Asian countries. In contrast to the much higher rates of female breast cancer (FBC), MBC rates are generally below 1 per 100,000 males per year [1, 2], which is equivalent to an overall female-to-male ratio of 122 [2]. In recent years, studies have reported differences in the biomarkers between MBC and FBC. Males exhibit a higher proportion of hormone receptor-positive breast cancers than do stage-, grade- and age-matched FBC patients [3, 4]. Over 90% of MBCs are estrogen receptor (ER)-positive, and 80–96% are progesterone receptor (PR)-positive [5, 6]. MBC tends to be more commonly characterized as ER and PR positive than FBC [7]. At the San Antonio Breast Cancer Symposium (SABCS, 2014), a recent study in the EORTC10085/TBCRC/BIG/NABCG program showed that 93% of 1822 MBC cases had high ER expression, 35% had high PR expression, and 9% had positive human epidermal growth factor receptor-2 (HER2) expression [8]. In our previous study [9], ER/PR positivity was also shown in more than 85% of MBC cases. However, post-menopausal FBC patients may more frequently have ER/PR-positive tumors compared with pre/peri-menopausal patients [7–9]. Because of the high ER expression in the tumor and low estrogen expression in the patient’s body, cases of FBC may be more recent in post-menopausal patients from a physiological perspective [10,11]. Some small-sample studies in the past have attempted to use endocrine therapy intended for post-menopausal FBC treatment to treat MBC based on these physiological similarities. However, thus far, these treatment attempts have failed to prove aromatase inhibitors (AIs) can be more effective in treating MBC than tamoxifen [10,11]. Therefore, tamoxifen remains commonly used to treat MBC patients.

Many studies have examined the differences in the survival rates between males and females; however, the results have been controversial [7,12,13]. Due to the large differences in hormone status between post-menopausal and pre/peri-menopausal patients, we do not believe that comparing MBC with FBC as a whole is reasonable; instead, we should compare MBC patients’ data separately with post-menopausal FBC and pre/peri-menopausal FBC. Thus, in this study, we attempt to investigate whether any differences in prognoses exist between MBC and FBC with different menstruation statuses.

## Methods

### Ethics statement

The Zhejiang Provincial Cancer Hospital Ethic Institution Office approved this study, and all participants voluntarily provided their written consent to participate in this clinical trial. This study strictly conforms to the principles outlined in the Declaration of Helsinki.

### Clinical materials

We selected 91 MBC cases without metastases (group M) that were diagnosed between January 2001 and January 2011 at Zhejiang Provincial Cancer Hospital. Each MBC case was paired with four FBC cases from the same period; two were pre/peri-menopausal (group A), and two were post-menopausal (group B). We used the NCCN guideline as the standard for determining menopause. In addition, in this experiment, the pre/peri-menopausal FBC patients who underwent ovarian function suppression (OFS) therapy or changed to AI treatment after becoming post-menopausal were included in the pre/peri-menopausal group (Group A). The clinical and pathological materials were collected. The following information was recorded: the patients’ ages, tumor sizes, lymph node metastases, clinical stages (based on the AJCC 7th Edition), and ER, PR, and HER2 expression. All patients underwent a standard operation and follow-up treatment that may have included the following: chemotherapy, endocrine therapy and radiotherapy (if necessary, according to the NCCN guideline). All of the ER/PR-positive MBC cases used tamoxifen as an endocrine therapy for at least 5 years.

In this study, all pathological and immunohistochemical materials (i.e., HE slices, immunohistochemical staining slices and paraffin-embedded slices) were assessed and confirmed by professional pathologists. ER and PR expression was measured using semi-quantitative cell nucleus scores. Cases that were 3+ by IHC or fluorescence in situ hybridization (FISH) were considered positive for HER2 expression. Cases that were IHC 1+/–or FISH—were considered negative for HER2 expression. Cases that were IHC 2+ were further tested by FISH to confirm the HER2 expression status.

### Follow-up data

All patients were interviewed via follow-up visits, telephone calls and direct mail questionnaires. The information collected in this study also included the first incidence of recurrence/metastasis (i.e., symptoms, signs or auxiliary examination results indicating recurrence or metastasis) and death related to breast cancer. Based on these data, we calculated the disease-free survival (DFS) and overall survival (OS) values for every patient.

### Statistical analysis

In this study, all data were analyzed using SPSS (v22.0). We used χ2 tests (and Fisher’s exact test, if necessary) to analyze numerical data. The survival times were compared among different groups using a Kaplan-Meier survival curve (log-rank test). Hazard ratios (HRs) of the DFS and OS values among different groups and all clinical and pathological factors were verified using a Cox proportional hazards model test.

## Results

### Clinical materials

In total, 91 cases of MBC and 364 cases of FBC were included in the analysis (Table 1). The mean age of group M was greater than that of group A (57.87 years and 43.73 years, respectively, p = 0.000). MBC cases had smaller tumors (average size 26.66 mm) than all FBC cases (average sizes for groups A and B: 30.71 mm, 29.70 mm, respectively, p<0.01). The clinical stages were similar among the three groups (p = 0.457). Group M cases were much more frequently ER positive than were groups A and B (80.22% vs 58.24% and 61.54%, p = 0.000 and p = 0.002, respectively); group M cases were also more frequently PR positive than were those of groups A and B (62.64% vs 41.21% and 45.60%, p = 0.001 and p = 0.008, respectively). Group M cases displayed much less HER2 expression than did those of groups A and B (6.59% vs 35.16% and 37.91%, p = 0.000 and p = 0.000, respectively). Among all groups, no HER2-uncertain cases were found. The adjuvant chemotherapy included of doxorubicin combined with cytoxan (AC*4), fluorouracil and doxorubicin combined with Cytoxan (FAC*6), AC-T and FAC-T according to the recurrence and metastasis risk degree of NCCN guideline and St.Gallen consensus. ER/PR+ male patients got tamoxifen as an endocrine treatment, while in ER/PR+ female of group B got arimedex (AI) and of group A got tamoxifen as endocrine treatment. Ten percentage of patients in group A changed into OFS or AI as continued endocrine treatment after tamoxifen.

**Table 1:** Clinical and pathological characteristics of groups M, A and B.

		Group M (N = 91)	Group A (N = 182)	Group B (N = 182)	p value
Age (years, mean±sd)		57.87±10.09	43.73±5.95	58.54±9.26	0.000* 0.593^§^
Tumor size (mm, mean±sd)		26.66±10.54	30.71±16.04	29.70±13.82	0.013* 0.045^§^
Clinical stage N (%)	I	25 (27.47)	48 (26.37)	38 (20.88)	0.910* 0.251^§^
	II	42 (46.16)	89 (48.90)	103 (56.59)	
	III	24 (26.37)	45 (24.73)	41 (22.53)	
ER N (%)	Positive	73 (80.22)	106 (58.24)	112 (61.54)	0.000* 0.003^§^
	Negative	18 (19.78)	76 (41.76)	70 (38.46)	
PR N (%)	Positive	57 (62.64)	75 (41.21)	83 (45.60)	0.001* 0.008^§^
	Negative	34 (37.36)	107 (58.79)	99 (54.40)	
HER2 N (%)	Positive	7 (7.69)	44 (24.18)	39 (21.43)	0.001* ^#^ 0.007^§^ ^#^
	Negative	84 (92.31)	138 (75.82)	143 (78.57)	

Group M, MBC; Group A, pre/peri-menopausal FBC; Group B, post-menopausal FBC; ER, estrogen receptor; PR, progesterone; HER2, human epidermal growth factor receptor-2.

* Group M compared with group A.

^§^ Group M compared with group B.

^#^ Fisher’s exact test.

#### Follow-up and prognoses

**Fig 1:**
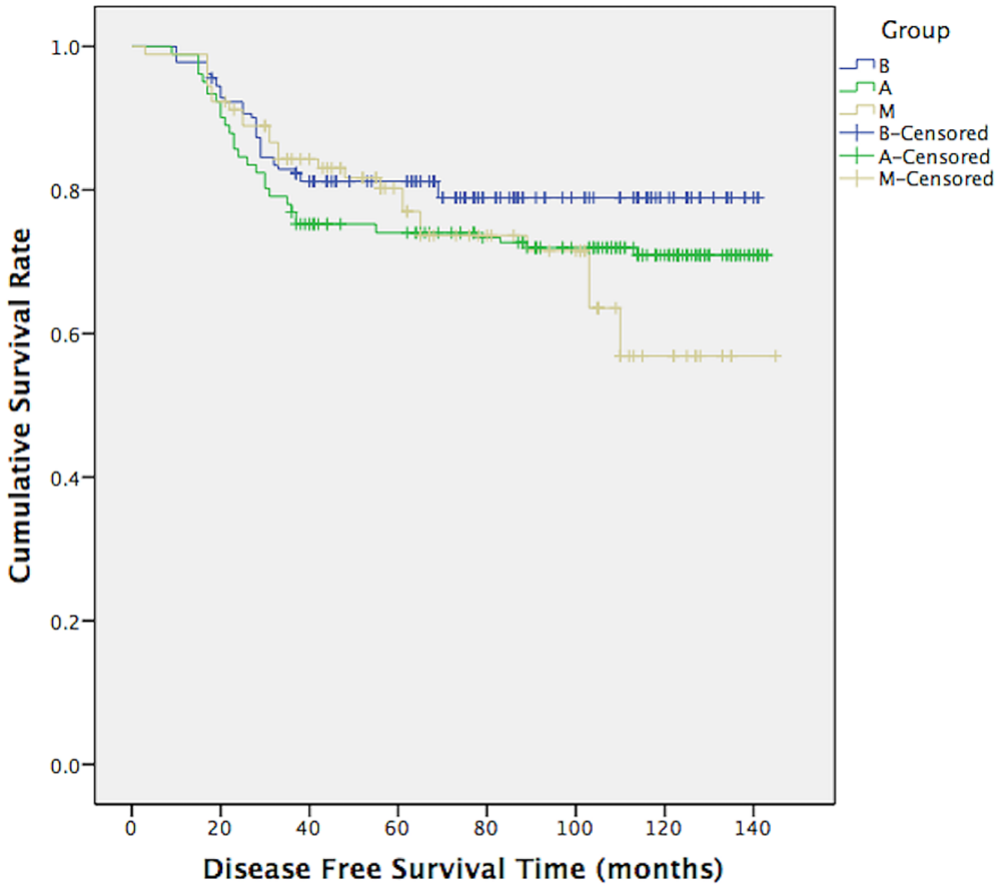
DFS of group M, group A and group B. Log-Rank test showed us the results: mean disease free survival (DFS) time of group M, group A and group B were 109.55 months, 110.87 months and 117.44 months. Comparing group M with group A, χ2 = 0.247, p = 0.619; comparing group M with group B, χ2 = 3.010, p = 0.083.

**Fig 2:**
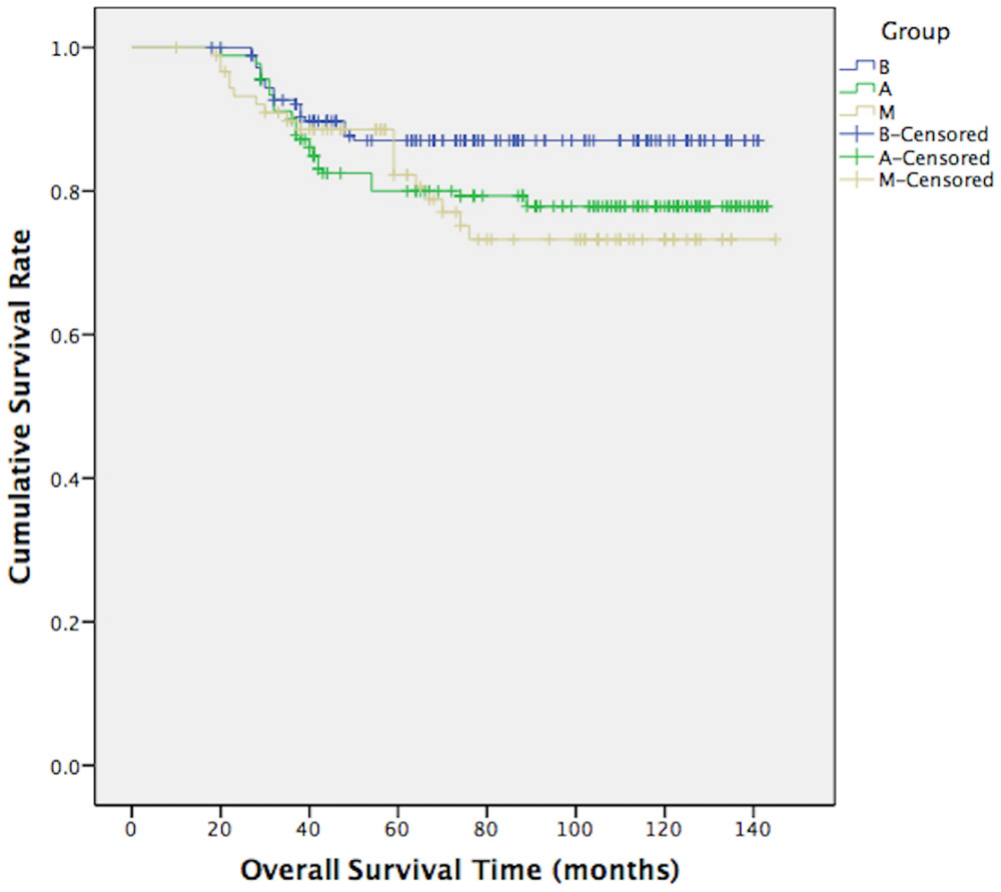
OS of group M, group A and group B. Log-Rank test showed us the results: mean overall survival (OS) time of group M, group A and group B were 119.26 months, 120.48 months and 127.32 months. Comparing group M with group A, χ2 = 0.139, p = 0.709; comparing group M with group B, χ2 = 4.141, p = 0.042.

The median follow-up period was 112 months (10–145 months). In total, 27 male patients in group M (29.67%) and 51 patients in group A (28.02%) had recurrence or metastasis events, which was a greater combined total than the 37 patients with such events in group B (20.33%). However, this result did not show any significant differences between the groups (comparing group M with groups A and B: p = 0.776, p = 0.086, respectively). In addition, no significant differences in DFS were observed between the different groups (log-rank test, group M vs groups A and B: p = 0.619 and p = 0.083, Fig 1); the p value was near 0.05 for the comparison between group M and group B. The 10-year OS rates of groups M, A and B were 79.1%, 79.1% and 87.9%, respectively. Group M had a significantly worse OS than group B (log-rank test, χ2 = 4.141, p = 0.042, Fig 2) but similar to that of group A (log-rank test, χ2 = 0.139, p = 0.709, Fig 2). The Cox proportional hazards regression model (Tables 2 and 3) indicated that pre/peri-menopausal FBC patients had DFS (HR = 0.706, p = 0.262) and OS (HR = 1.029, p = 0.941) values similar to those of MBC patients, whereas post-menopausal FBC patients had better DFS (HR = 0.454, p = 0.004) and OS (HR = 0.353, p = 0.003) values than MBC patients.

**Table 2:** Cox proportional hazards regression model test for the DFS values of all patients.

	B	Wald	p value	*Exp* (B)	95% CI of *Exp* (B)	
					Lower	Upper
Age	0.017	1.954	0.162	1.017	0.993	1.042
Size	0.010	2.817	0.093	1.010	0.998	1.022
Clinical stage	-	19.177	0.000	-	-	-
ER	1.355	33.809	0.000	3.878	2.456	6.124
PR	0.393	3.179	0.075	1.482	0.962	2.283
HER2	-0.288	1.850	0.174	0.749	0.495	1.136
Group A vs Group M	-0.348	1.258	0.262	0.706	0.385	1.297
Group B vs Group M	-0.790	8.382	0.004	0.454	0.266	0.775

Group M, MBC; Group A, pre/peri-menopausal FBC; Group B, post-menopausal FBC; ER, estrogen receptor; PR, progesterone; HER2, human epidermal growth factor receptor-2.

**Table 3:** Cox proportional hazards regression model test for the OS values of all patients.

	B	Wald	p value	*Exp* (B)	95% CI of *Exp* (B)	
					Lower	Upper
Age	0.044	8.549	0.003	1.045	1.015	1.076
Size	0.014	4.108	0.043	1.014	1.000	1.028
Stage	-	5.140	0.077	-	-	-
ER	1.706	31.799	0.000	5.504	3.043	9.957
PR	0.688	5.442	0.020	1.990	1.116	3.547
HER2	-0.192	0.530	0.467	0.825	0.492	1.385
Group A vs Group M	0.029	0.006	0.941	1.029	0.484	2.189
Group B vs Group M	-1.042	9.123	0.003	0.353	0.180	0.694

Group M, MBC; Group A, pre/peri-menopausal FBC; Group B, post-menopausal FBC; ER, estrogen receptor; PR, progesterone; HER2, human epidermal growth factor receptor-2.

## Discussion

The majority of previous studies have indicated that ER/PR expression is higher in MBC than FBC [1,3,14]. In this study, we found a similar result. Among the MBC cases, 80.22% were ER positive, and only 7.69% were HER2 positive.

Some reports have also shown that hormone receptor status and HER2 expression strongly influence MBC prognosis and may lead to different prognoses between MBC and FBC [5,15]. In addition, therapies for MBC and FBC have varied over the last decade, which may also be a factor that has confounded the prognoses in many studies. Thus, in this study, we included female and male cases from the same period and matched our female and male subjects.

MBC is recognized as having a worse overall prognosis than female breast cancer [16]; however, when the cancer stage, patients’ ages and prognostic factors are controlled, the prognoses are similar [15,17,18]. According to Wang-Rodriguez et al [15], the clinical stage (irrespective of nodal status or tumor size) is the single most significant prognostic factor. Our results showed that MBC patients had smaller tumors than FBC patients did; based on clinical experiences, we observed that males have thinner mammary glands and, thus, tend to have a higher probability of finding palpable tumors at an early stage than do females. In our opinion, the clinical stage is important to both MBC and FBC but may not contribute to a significant difference in OS in the total population of males and females, as shown in our Cox regression model analysis (p = 0.077). The p value was nearly 0.05; thus, a study with a greater number of paired cases is needed to confirm this result.

Few studies have focuses on an important question based on the lower estrogen level of the body and higher ER expression in the tumor in males than in females, namely, whether MBC patients can obtain similar survival benefits from endocrine therapy as do FBC patients, particularly the post-menopausal female population. As is known, post-menopausal FBC patients also have low estrogen levels and high ER expression and can thus be recognized as a different population than pre/peri-menopausal FBC patients. Hence, in our opinion, when comparing MBC and FBC regarding prognoses such as DFS and OS, we should separate FBC patients into different groups according to their menstruation status. Log-rank analysis results showed that MBC had DFS and OS values similar to those of pre/peri-menopausal FBC but worse compared with those of post-menopausal FBC. The Cox regression model analysis also showed that ER positivity and post-menopausal status were both important protective factors for OS. We can conclude that ER-positive men and women can obtain a prognostic benefit from endocrine therapy and that post-menopausal women obtain better results due to the common use of AIs among female patients. Based on this result, we hope that large clinical trials for using AIs to treat MBC can provide us a clear answer regarding whether MBC patients can benefit more from AIs than from tamoxifen.

A number of studies have reported varying rates of HER2 positivity in MBC [8,15,19–23]. Our study revealed that an extremely low proportion of MBC cases were HER2 positive (7.69%), which is in agreement with previous studies. Thus, HER2 expression in MBC remains controversial, and clarification of this issue requires further investigations involving large sample sizes. From 2001 to 2010 in China, few people could afford trastuzumab for anti-HER2 cancer treatment; thus, in our research, we collected FBC cases within the same period as each MBC case as a way to minimize the number of varied factors. Our result showed that MBC patients had much lower HER2 positivity than did FBC patients (7.69% vs 24.18% and 21.34%). Because of such a low percentage of HER2 positive in MBC patients, we could not judge the effect of anti-HER2 treatment in prognosis of MBC comparing with FBC; we still need more data and more samples to exploring this answer.

## Conclusion

Based on our study, on the one hand, we found that the ER/PR expression in MBC notably differs from that in FBC. On the other hand, the menstruation status had an important influence on hormone levels and on ER/PR expression, which separates FBC cases into two groups. Thus, comparing the prognosis of MBC with FBC according to the different menstruation statuses of women is reasonable.

Our study results confirmed that no absolute evidence indicates that MBC has a worse prognosis than total FBC but rather that MBC specifically has a worse prognosis than the post-menopausal FBC subgroup. Because the sample employed in our study was small and because the therapy was not controlled accurately, we believe that further research investigating the role of treatment factors on the prognoses of MBC and FBC is essential, particularly for endocrine therapy.
